# Identification of *Delia* spp. (Robineau-Desvoidy) (Diptera, Anthomyiidae) and its cruciferous hosts in Mexico

**DOI:** 10.3897/zookeys.964.53947

**Published:** 2020-08-27

**Authors:** Ricardo Meraz-Álvarez, Néstor Bautista-Martínez, Carlos Patricio Illescas-Riquelme, Héctor González-Hernández, Jorge Manuel Valdez-Carrasco, Jade Savage

**Affiliations:** 1 Colegio de Postgraduados, Posgrado en Fitosanidad-Entomología y Acarología, Carretera México-Texcoco Km. 36.5, Montecillo, Texcoco 56230, Estado de México, México Colegio de Postgraduados Texcoco Mexico; 2 Centro de Investigación en Química Aplicada, Blvd. Enrique Reyna Hermosillo No.140 C.P. 25294 Saltillo, Coahuila, México Centro de Investigación en Química Aplicada Saltillo Mexico; 3 Biological Sciences, Bishop’s University, Sherbrooke, Quebec, J1M 1Z7, Canada Bishop’s University Sherbrooke Canada

**Keywords:** Male genitalia, molecular identification, soil pests, root damage, root maggots, weeds, wild hosts

## Abstract

Soil pests of cruciferous crops in Mexico have been gaining importance in recent years; such is the case of *Delia* spp. (Robineau-Desvoidy) (Diptera, Anthomyiidae), of which, to date, there are no studies on the correct identification of associated species, as well as the range of hosts. In an integrated pest management program, it is essential to know this information to design and implement adequate phytosanitary measures. Plants infested by *Delia* spp. were collected in the states of Guanajuato, Puebla, and Mexico from June to November 2017 and March to December 2018 in commercial plantations of cruciferous crops (*Brassicaoleracea* L. var. *italica*, *botrytis* and capitata), *B.napus* L., and *Raphanussativus* L.) as well as some cruciferous weeds (*R.raphanistrum* L., *Sisymbriumirio* L., *B.campestris* L., *Capsellabursa-pastoris* L., and *Lepidiumvirginicum* L.) in the edges of these crops. The two species found in this study, *Deliaplanipalpis* (Stein) and *Deliaplatura* (Meigen), identified using male genitalia was corroborated by molecular techniques. Both species emerged from all the sampled hosts, except for *C.bursa-pastoris* and *L.virginicum*. The association of the two species in cruciferous crops and weeds, provides valuable information for the management of these insects not only in cruciferous crops but other ones that are strongly attacked by *D.platura*.

## Introduction

The family Anthomyiidae, commonly called root maggots (Huckett 1965), is a large group of flies of the dipteran clade Muscoidea which also includes house flies (Muscidae), latrine flies (Fannidae), and dung flies (Scathophagidae) (Ding et al. 2015; Kutty et al. 2019). The larvae are mainly phytophagous or saprophagous. They have been found in stems, roots, floral heads, and foliage of live plants as well as plants in process of decomposition. Some are scavengers or coprophagous in excrement of birds and other animals. Others are tenants, diners, or parasites in nests of bees, solitary wasps, rodents, and land turtles. They are also found on beaches where they feed on seaweeds and near freshwater ponds, or streams (Huckett 1987; Smith 1989). They can also be omnivorous; certain species are known to be endoparasitoids of grasshoppers and kleptoparasitoids in hymenopteran nests (Suwa 1974; Gilbert and Jervis 1998), and there are predators of simulid larvae (Ackland and Werner 2006).

According to Michelsen (2014), nearly 2 000 species are known worldwide, but undoubtedly there are more waiting to be described (Smith 1989). Although they are distributed the world over, this family is better represented in temperate regions, especially in the Holarctic region. Almost 600 species belonging to 50 to 60 genera are known in the Nearctic region and a similar number is known in the Palaearctic region (Huckett 1987; Smith 1989).

From an economic standpoint, some species are phytophagous and feed on live plant tissues (Hill 1987) of food crops, ornamentals, weeds (Huckett 1987), and forest trees (Suwa 1974; Turgeon and Sweeney 1993). Some family members are significant agricultural pests, particularly those that belong to the genus *Delia* (Robineau-Desvoidy), such as *D.radicum* (Linnaeus), *D.platura* (Meigen), *D.planipalpis* (Stein), *D.florilega* (Zetterstedt), *D.floralis* (Fallén), and *D.antiqua* (Meigen) (Savage et al. 2016). Also included are cereal sprout flies (*D.coarctata* (Fallen), *D.arambourgi* (Seguy), *D.flavibasis* (Stein)) (Macharia and Mueke 1986), and miners (*D.echinata* (Seguy), *D.cardui* (Meigen), and *D.brunnescens* (Zetterstedt)) (Hill 1987). Certain *Delia* species have a relatively small range of hosts. *Deliaradicum* and *D.antiqua*, for example, attack only plants of the family Brassicaceae and *Allium* spp., respectively. However, *D.platura* and *D.florilega* have a wide range of hosts including species of Brassicaceae and *Allium* spp. in decomposing process as well as legumes, Cucurbitaceae, and some cereals (Howard 1994). In general, they attack a larger diversity of plant species than their common name indicates (Brooks 1951).

The damage *Delia* spp. cause to vegetables, cereals, ornamentals, and forest species is considerable. An example of this is *D.radicum*, one of the most studied species and considered the primary pest of several crops of the Brassicaceae family in temperate latitudes (35–60°N) of North America, Europe and Asia (Dixon et al. 2014). In Canada, where most of the provinces raise crucifers such as cabbage, cauliflower, broccoli, and rutabaga (*Brassicanapus* var. napobrassica (L.) Rchb.), the problem becomes acute because there are few authorized pesticides, such as diazinon and chlorpyrifos (van Herk et al. 2017). Additionally, resistance to chlorpyrifos is confirmed in areas where rutabaga is cultivated (Blackshaw et al. 2012) and where there are high concentrations of pesticide residue in aquifers (Joseph and Zarate 2015).

In Mexico, 67.7% of the total income from export of produce is earned by 20 crops, among which is broccoli, cultivated mainly in Guanajuato (24 886 ha), Puebla (2 772 ha), and Michoacán (2 225 ha). Mexico is considered the fifth world producer of broccoli and cauliflower (SIAP 2018). In the main crucifer-producing regions, the economically important pests are diamondback moth (*Plutellaxylostella* (Linnaeus)) (Lepidoptera, Plutellidae), cabbage looper (*Trichoplusiani* (Hübner)) (Lepidoptera, Noctuidae), cabbage worm (*Copitarsiadecolora* (Guenée)), and cabbage aphid (*Brevicorynebrassicae* (Linnaeus)) (Hemiptera, Aphididae). Contamination by several biological stages of these pests, as well as their excretions, affects the quality or health of the produce. There are also other secondary pests that, if they are not adequately managed, can have a negative impact on yield and quality of the harvest (Barrios-Díaz et al. 2004; Suárez-Vargas et al. 2006; Santoyo-Juárez and Martínez 2011; Bujanos-Muñiz et al. 2013a, 2013b).

In recent years in different regions of the country, major outbreaks of root maggot (*Delia* spp.) have occurred in crucifers. However, the identification of these insects has not been sufficiently supported, and identification has only been to the genus level. There are reports from the state of Guanajuato which mentioned flies of the genus *Hylemia* (= *Delia*) associated with maize and beans, as well as with crucifers (Marín-Jarillo 2001). In the region of Acatzingo, Puebla, in the 2000 spring-summer crop cycle, the pest was detected in a cabbage crop and identified as *Hylemia* sp. (= *Delia*) (Barrios-Díaz et al. 2004).

Because integrated management of any pest requires reliable diagnosis and, given the economic importance, the difficulty of identifying this group of insects and the lack of research to date in the country, this study posed the following objectives: to identify the *Delia* species complex associated with broccoli (*B.oleracea* var. *italica*), cabbage (*B.oleracea* var. *capitata*), and cauliflower (*B.oleracea* var. *botrytis*) crops principally and to determine their range of cruciferous hosts as well as the type of damage they cause.

## Materials and methods

*Delia* species for identification were collected in cultivated and wild crucifers from June 2017 to December 2018 in the states of Guanajuato, Puebla, and Mexico. The crops included in the collections were broccoli (*B.oleracea* var. *italica*), cabbage (*B.oleracea* var. *capitata*), and cauliflower (*B.oleracea* var. *botrytis*), as well as turnip (*B.napus* L.), radish (*Raphanussativus* L.), and other wild crucifers such as wild radish (*Raphanusraphanistrum* L.), field mustard (*Brassicacampestris* L.), London rocket (*Sisymbriumirio* L.), shepherd’s purse (*Capsellabursa-pastoris* L.), and Virginia pepperweed (*Lepidiumvirginicum* L.) For the cultivated crucifers, 10–15 plants with symptoms of wilting were selected in each lot, as well as less developed contiguous plants and some apparently health plants. Wild crucifer plants were selected at random within and on the outer edges of commercial crops; these plants generally did not show wilting symptoms, and the sample size varied from 5 to 20 plants, depending on their abundance in the crop as a consequence of weed control. The plants on which *Delia* larvae were detected were extracted intact together with the soil adhered to the roots. Later, all the plants collected from the same farm were grouped and placed into 2–3 L plastic bags and labeled with locality, date, and host, separating cultivated from wild hosts. The age of the crops from which infested material was collected ranged from 20 to 70 days after transplant to the field. In the case of wild crucifers, the specimens collected ranged in maturity from seedlings to plants with flowers and seeds. The material was transported to the Laboratory of Agricultural Entomology of the Colegio de Postgraduados, Campus Montecillo, Texcoco, State of Mexico, and confined. The samples were kept in a rearing chamber at a temperature of 26±2 °C, 60±20% relative humidity, and photoperiod of 12:12 (light:dark) until adult emergence. As the adults emerged, they were separated by sex and morphotype for each sample.

### Species identification

Morphological identification of the specimens (including the traits of the male genitalia) was conducted in the Laboratory of Agricultural Entomology of the Colegio de Postgraduados, Campus Montecillo. The keys and illustrations by Darvas and Szappanos (2003) and Savage et al. (2016) were used to differentiate sexes and to identify species; the distance between the eyes (holoptic males and dichoptic females) and the chaetotaxy of the hind femur were used. Images were taken with a Photomicroscope III Carl Zeiss (Carl Zeiss, Germany). To confirm the identity of the collected species, DNA barcodes (Hebert et al. 2003) were used. DNA was extracted from the mitochondrial gene of the cytochrome c oxidase subunit I (COI) (Folmer et al. 1994) of 25 adult specimens (19 males and six females) and amplified. The sequences of this material can be consulted in Barcode of Life Data System (BOLD) (http://www.barcodinglife.org) in the public database *Delia* of Mexico (https://doi.org/10.5883/ds-domex) and all sequences were deposited in GenBank (accession numbers MT888006–MT888030). The sequences of at least 550 base pairs were grouped using the BOLD aligner. Intraspecific and interspecific distances were calculated in BOLD using the distance model Kimura 2 parameters (K2P) (Kimura 1980).

The specimens are deposited in the National Center for Phytosanitary Reference, Division of Plant Health, SENASICA, Tecámac, State of Mexico. Adult and larval specimens were also provided to the entomological collection of the Colegio de Postgraduados, Campus Montecillo. Moreover, the specimens used for molecular identification are in the insect collection of Bishop’s University, Quebec, Canada.

## Results

Tables 1 and 2 show the number of emerged adults in each of the samples collected in cultivated and wild cruciferous, respectively. *Deliaplanipalpis* and *D.platura* emerged from both cultivated and wild hosts. The number of adults of *D.planipalpis* was greater than *D.platura* in 89% and 88% of the cases, respectively. Both species emerged from all the hosts except from *C.bursa-pastoris* and *L.virginicum* where no damage from larvae of *Delia* spp. was observed when these were collected.

**Table 1.** Number of *Deliaplanipalpis* and *D.platura* adults emerged in cultivated crucifers.

Collection site	Crop	*Deliaplanipalpis*			*Deliaplatura*			Collection date
		♀♀	♂♂	Total	♀♀	♂♂	Total	
San Felipe Tenextepec, Tepeaca, Puebla 19°01'39.80"N, 97°52'12.28"W	Cauliflower	3	5	8	2	1	3	31-V-2017
	Broccoli	1	2	3	1	3	4	
	Broccoli	16	12	28	0	0	0	25-VIII-2017
	Turnip	15	9	24	1	0	1	13-IV-2018
	Broccoli	4	1	5	0	0	0	12-VII-2018
	Broccoli	4	9	13	0	0	0	8-XI-2018
San Mateo Parra, Tepeaca, Puebla 18°59'35.98"N, 97°51'43.20"W	Broccoli	2	2	4	1	0	1	12-VII-2017
Guadalupe Calderón, Tepeaca, Puebla 18°57'27.35"N, 97°50'43.51"W	Cauliflower	0	3	3	0	1	1	12-VII-2017
Acatzingo, Puebla 18°58'30.72"N, 97°47'53.55"W	Cabbage	2	4	6	3	0	3	12-VII-2017
		25	17	42	11	15	26	18-X-2017
		0	0	0	2	4	6	13-IV-2018
Tepeaca, Puebla 19°00'04.9"N, 97°53'12.8"W	Cabbage	5	7	12	0	0	0	29-IX-2018
	Cabbage	12	12	24	0	2	2	22-XI-2018
Los Reyes, Tepeaca, Puebla 18°57'27.18"N, 97°50'50.24"W	Radish	19	35	54	1	1	2	6-XII-2018
Montecillo, Texcoco, Estado de México 19°28'10"N, 98°54'00.81"W	Radish	1	0	1	0	0	0	27-IV-2018
	Radish	4	2	6	3	1	4	11-V-2018
	Radish	5	3	8	3	2	5	19-V-2018
Dolores Hidalgo, Guanajuato 21°09'52"N, 100°57'18"W	Broccoli	4	3	7	1	0	1	8-V-2018
San Luis de la Paz Guanajuato 21°19'23"N, 100°33'22"W	Broccoli	6	4	10	2	1	3	6-IV-2018
San Diego de la Unión, Guanajuato 21°24'30.4"N, 100°45'19.3"W	Broccoli	7	4	11	1	1	2	25-X-2018
	Broccoli	13	11	24	0	0	0	4-XII-2018

**Table 2.** Number of *Deliaplanipalpis* and *D.platura* adults emerged in wild crucifers.

Collection site	Host	*Deliaplanipalpis*			*Deliaplatura*			Collection date
		♀♀	♂♂	Total	♀♀	♂♂	Total	
Acatzingo, Puebla 18°58'30.72"N, 97°47'53.55"W	*R.raphanistrum*	2	1	3	0	0	0	12-VII-2017
	*S.irio*	3	1	4	0	1	1	12-VII-2017
	*R.raphanistrum*	21	20	41	0	1	1	22-XI-2018
San Felipe Tenextepec, Puebla 19°01'39.80"N, 97°52'12.28"W	*R.raphanistrum*	0	0	0	1	2	3	12-VII-2017
	*R.raphanistrum*	20	37	57	2	6	8	8-XI-18
Los Reyes, Tepeaca, Puebla 18°57'27.18"N, 97°50'50.24"W	*R.raphanistrum*	21	23	44	0	0	0	6-XII-2018
Montecillo, Texcoco, Estado de México 19°28'10"N, 98°54'00.81"W	*R.raphanistrum*	3	1	4	11	8	19	26-III-2018
	*C.bursa-pastoris*	0	0	0	0	0	0	26-III-2018
	*S.irio*	5	3	8	1	3	4	29-III-2018
	*B.campestris*	7	2	9	0	0	0	29-III-2018
	*R.raphanistrum*	3	5	8	1	2	3	1-IV-2018
	*L.virginicum*	0	0	0	0	0	0	1-IV-2018
	*B.campestris*	1	0	1	1	0	1	3-IV-2018
	*C.bursa-pastoris*	0	0	0	0	0	0	1-IV-2018
	*L.virginicum*	0	0	0	0	0	0	1-IV-2018
	*S.irio*	2	5	7	0	0	0	11-IV-2018
	*R.raphanistrum*	0	0	0	1	0	1	16-IV-2018
	*B.campestris*	17	10	27	2	1	3	27-IV-2018
	*S.irio*	1	2	3	2	0	2	6-V-2018
	*S.irio*	0	2	2	0	0	0	6-V-2018
	*R.raphanistrum*	6	7	13	0	0	0	20-X-2018
	*C.bursa-pastoris*	0	0	0	0	0	0	20-X-2018
	*L.virginicum*	0	0	0	0	0	0	20-X-2018

## Discussion

**Figure 1.**
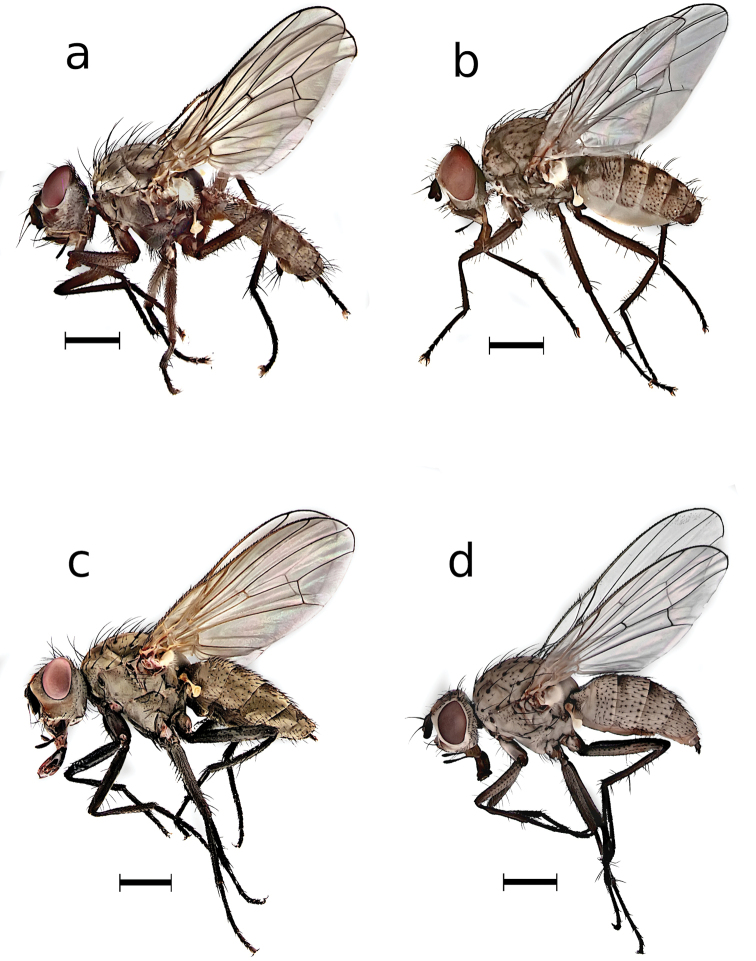
**a** male *Deliaplanipalpis***b** male *Deliaplatura***c** female *Deliaplanipalpis***d** female *Deliaplatura*. Scale bars: 1 mm.

**Figure 2.**
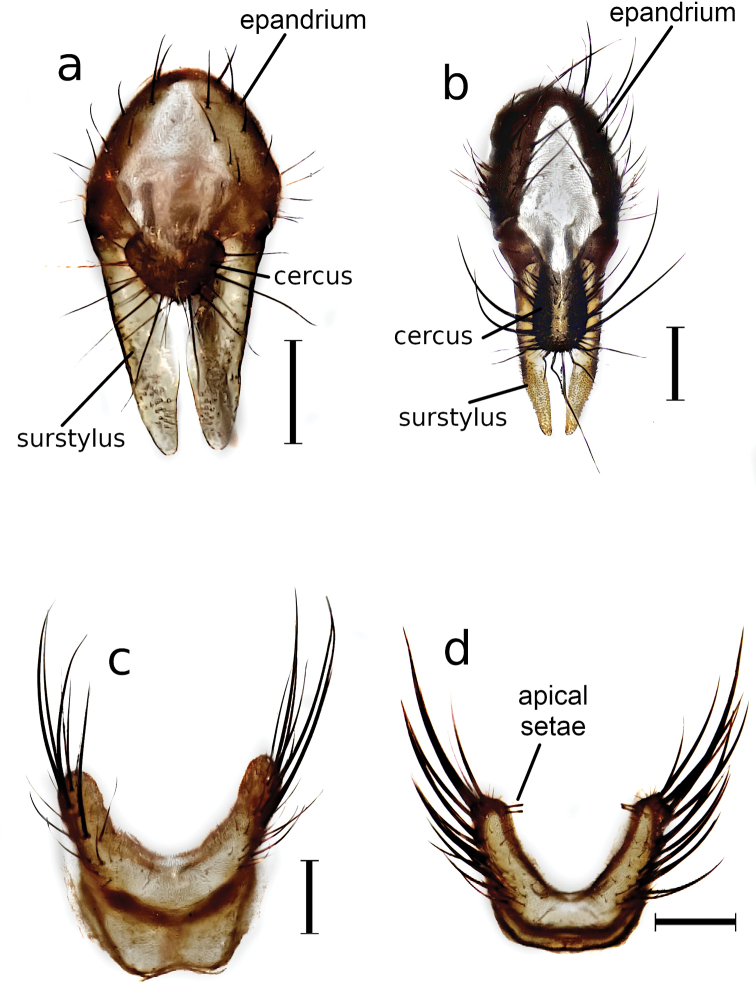
Male genitalia. **a** terminalia of *Deliaplanipalpis***b** terminalia of *D.platura***c** abdominal sternite V of *D.planipalpis***d** abdominal sternite V of *D.platura*. Scale bars: 200 nm.

The specimens were identified as *Deliaplanipalpis* (Stein) and *Deliaplatura* (Meigen) (Fig. 1) using male genitalia as the principal reference since they are the fundamental identification tool for species of the family Anthomyiidae (Darvas and Szappanos 2003). The extracted male genitalia of *D.planipalpis* and *D.platura* male genitalia are similar to those illustrated by Wang et al. (2014), Savage et al. (2016), and Darvas and Szappanos (2003). In addition, there are morphological differences that are highly useful in separating these two species. In the terminalia of *D.planipalpis*, the short, armored cercus with radially arranged setae does not extend beyond the tip of the surstylus, whose arms are thinner at the basal and apical parts than in the middle. The setae of the epandrium are short and sparse. In contrast, the cercus in *D.platura* is elongated and oval, with numerous setae directed toward the front and upward; in length the setae can reach the tip or extend beyond the tip of the surstylus arms, and extend approximately to mid-length of the surstylus between the arms, which tend to be narrower at the apex and have short setae on the lateral margins. In *D.planipalpis* abdominal sternite V lacks the pair of setae at the apex of each of the arms, as in *D.platura* (Fig. 2).

**Figure 3.**
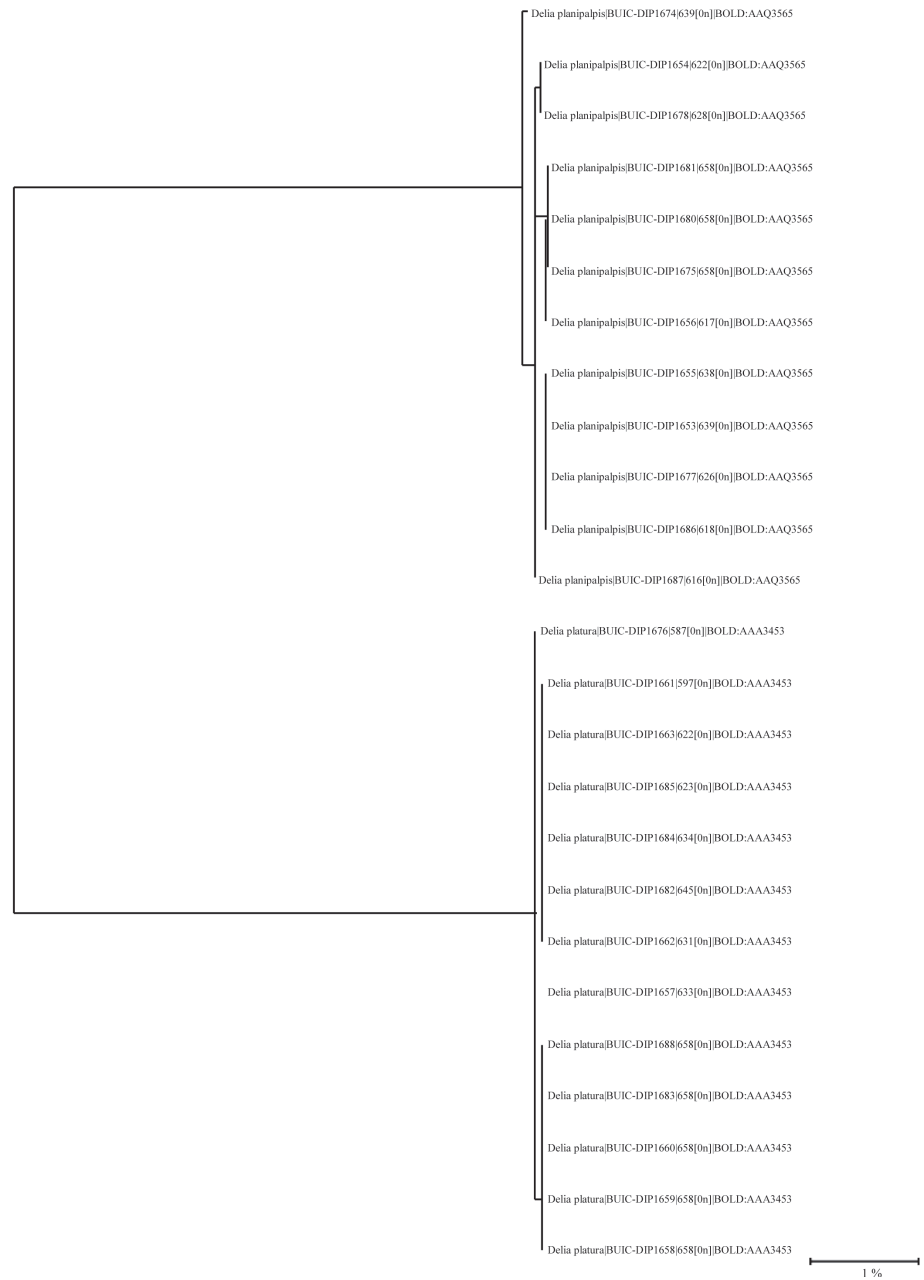
Phylogenetic tree (based on the K2P distance model) for 25 COI sequences (minimum 550 bpm, 0 bp ambiguous) of *Delia* specimens. The line includes species name, sample identification by BOLD, sex, and barcode index number (BIN).

The results of the DNA barcodes were congruent with the morphology and also indicated that all the specimens sequenced for *D.platura* belong to BOLD:AAA3453, one of the two different barcode index numbers (BIN) for this species. This population is found almost exclusively in the New World (Savage et al. 2016). The material of each species formed a compact cluster in the phylogenetic tree (Fig. 3), with each belonging to a different BIN (Ratnasingham and Hebert 2013). The greatest interspecific distance was 10.49% and the greatest intraspecific distance was 0.34% for *D.planipalpis* and 0% for *D.platura*.

This is the first report of species-level identifications of *Delia* in commercial crucifer crops and wild hosts in Mexico. It is supported by images of adult male genitalia and corroborated by COI gene sequence data. However, there are also external morphological traits that are very useful for initial diagnosis, such as the array of setae along the hind femurs of males and females (Savage et al. 2016) and chaetotaxy in general. Nevertheless, identification based solely on chaetotaxy has generally not been sufficient and is often the cause of confusion in the literature; there are many questionable records (Darvas and Szappanos 2003). It is possible to separate the two species identified here because all their biological stages exhibit morphological differences, unlike other species whose immature states are morphologically indistinguishable. For example, in some regions, *D.planipalpis* is confused with *D.radicum*, and *D.platura* is confused with *D.florilega* (Savage et al. 2016), and thus, the presence of one species might be overlooked because of a mistaken identification. In general, the family Anthomyiidae is considered a taxonomically complex insect group because the traits used to differentiate sexes and species are not always constant (Colyer and Hammond 1968).

For this reason, it is understandable that little or no research on this insect group has been done in Mexico; even the most common *Delia* pest is difficult to identify without adequate training. Furthermore, the challenge becomes greater when dealing with females or immature specimens that lack the characteristics for diagnosis (Savage et al. 2016). Females cannot be identified without an appropriate key (Darvas and Szappanos 2003). Finally, few people work with this type of pest, which results in national collections with vague identifications and a scarcity of well-preserved specimens that could contribute to the knowledge of their distribution, hosts, and dates of appearance, among other data.

*Deliaplanipalpis* and *D.platura* emerged in both cultivated (Table 1) and only from wild (Table 2) hosts *R.raphanistrum*, *B.campestris*, and *S.irio*. This coincides with information presented by Savage et al. (2016), who mentioned that *D.platura* is generally found in infestations together with other species of *Delia* and their association depends on the host. For example, in bean seed in Canada, it is associated with the seed-infesting fly *D.florilega*. In our study, more *D.planipalpis* adults emerged than *D.platura* in cultivated (Table 1) and wild (Table 2) crucifers; *D.planipalpis* is catalogued as a phytophagous species that mainly attacks radish (Kelleher 1958), unlike *D.platura* that is phytophagous only under certain circumstances causing damage to roots and often appears in small numbers in conjunction with other phytophagous species (Brooks 1951). In this respect, combined infestations have been reported of *D.platura*/*D.antiqua* (Finlayson 1956) and *D.platura*/*D.florilega* (Savage et al. 2016) in onion. Finch (1989) mentions that *D.platura* is not a primary species and only invades seeds when the seed coat has been infested by pathogens before germinating.

### Damage

**Figure 4.**
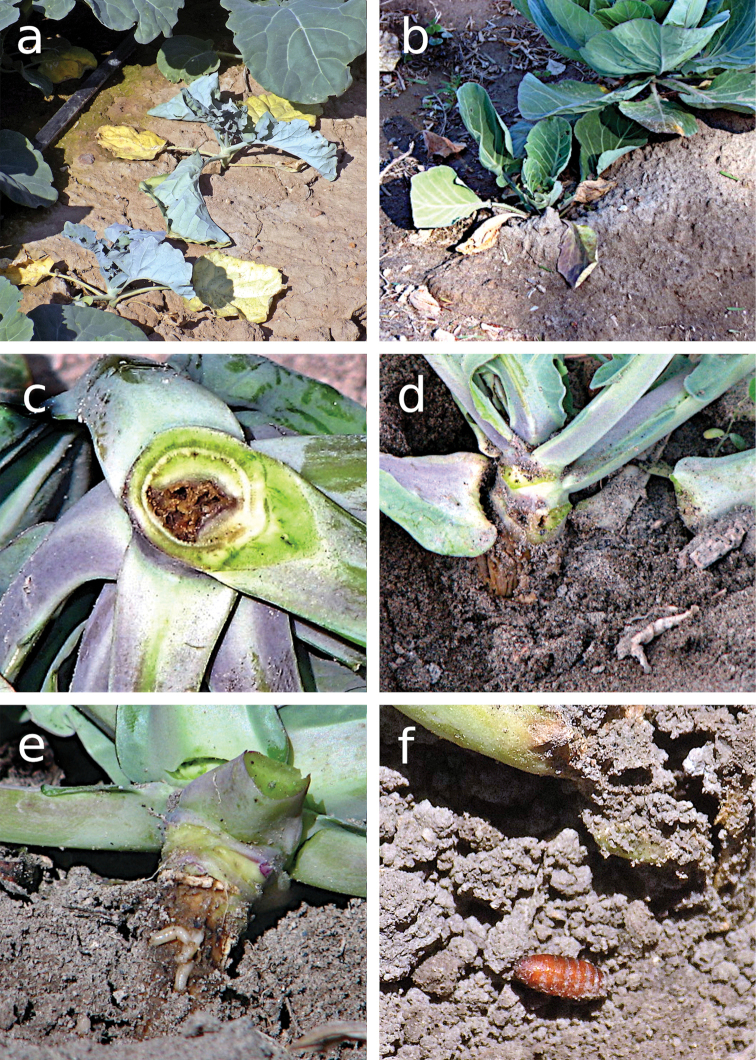
Damage caused by *Deliaplanipalpis* and *D.platura*. **a** broccoli plants damaged by *Delia* spp. larvae **b** cabbage plant with yellow wilted basal leaves **c** main stem of a cabbage plant with galleries **d** cabbage plant with holes where larvae exited **e***Delia* spp. larvae feeding superficially on the stem of a cabbage plant **f***Delia* spp. pupa at the site where plant was extracted.

Female *Delia* spp. oviposit at the base of the plant stem and in the surrounding soil. Once the larva emerges, it feeds on external tissue of the stem before penetrating it or the basal leaves. The level of damage caused by *Delia* spp. larvae is in function of plant age: between 10 and 30 days after transplant, the damaged plants exhibit symptoms similar to those caused by water deficit (Fig. 4a, b), and they may die or have delayed growth with consequentially poor-quality inflorescence. *Delia* spp. larvae in *B.oleracea* (var. *italica*, *capitata*, and *botrytis*) crops are generally found at the base of the plant feeding on the root crown, damaging the main stem and the root system and causing the plant’s death or notably delaying its growth. Moreover, galleries resulting from larva feeding can be seen in the main stem as well as orifices through which third instar larvae exit to pupate in the soil (Fig. 4c, d). Pupae can be observed at the site where the attacked plant is extracted (Fig. 4f) and in the substrate adhered to the roots. Older plants, more than 30 days after transplant, can tolerate the damage caused by feeding larvae, reflecting plant vigor. According to Wheatley and Finch (1984), crops that grow vigorously can bear large populations without showing symptoms, although attacked plants will be smaller, and the quality of the final product will be poorer. This occurs when *Delia* spp. larvae feed superficially on the external tissues of the main stem (Fig. 4e) and penetrate basal leaves, which become yellow and wilted (Fig. 4b).

In a broccoli field nearing harvest time, damage caused by third instar *D.planipalpis* larvae was observed at the base of the upper stratum leaves, very close to the floret. With this damage, the leaves will fall off, and under conditions of high relative humidity, other insects and saprophagous organisms enter.

Another type of damage caused in radish and turnip is the formation of galleries in the edible part. It is common that the damage in these hosts begins in the plant core and continues into the harvestable part. Although this type of damage does not generally cause plant death, the produce is not suitable for commercial sale. Death of radish plants occurs when infestations are high, or the plants are still small.

During field observations, we were able to confirm the presence of adult *D.planipalpis* and *D.platura* on the edges of the crop fields. However, this is not necessarily indicative of significant damage to the crop caused by larvae. Savage et al. (2016) mention that some *Delia* species can be highly abundant as adults, but they are rarely involved in crop damage. In a cabbage field close to harvest we observed adults on the periphery of the crop; they likely came from neighboring crucifer crops and wild crucifers of the area. These observations coincide with Hawkes (1972), who reported that adult *Erioischiabrassicae* (Bouché) (= *Deliaradicum* L.), once they locate the crop, remain on the edges during the morning and in the afternoon move into the crop to oviposit and finally return to the edges of the crop at sunset.

## Conclusions

Two species of *Delia* were identified, *Deliaplanipalpis* and *D.platura*, which were found associated with broccoli (*B.oleracea* var. *italica*), cabbage (*B.oleracea* var. *capitata*), and cauliflower (*B.oleracea* var. *botrytis*), as well as in radish (*R.sativus*) and turnip (*B.napus*). The extent of damage caused by *Delia* spp. larvae depends on plant age and crop type. For example, in *B.oleracea*, *Delia* spp. can cause plant death, delay growth, or make the produce unfit for commercialization because of damage caused to the harvestable part, as also for *R.sativus* and *B.napus*.

*Deliaplanipalpis* and *D.platura* larvae generally feed on the same plant and pupate in the soil near the plant root or in the same germination substrate that remains adhered to the roots. In the wild crucifers *R.raphanistrum*, *B.campestris*, and *S.irio*, which are alternate hosts, it is also common to find both *Delia* species feeding on the same plant. However, they do not cause plant death, even in the seedling stage.

Given the field observations, it is likely that *D.planipalpis* is the species that first invades healthy plants and, as damage by the feeding larvae progresses, *D.platura* is later attracted by the volatiles emitted by the plant. Nevertheless, study is needed to determine the possible volatile compounds emitted during decomposition of plant tissue caused as by the feeding of *D.planipalpis* larvae and to identify the moment when *D.platura* arrives. This kind of basic information is useful to design specific phytosanitary measures to control *D.planipalpis*, not only on cruciferous crops, but even on other crops that are strongly attacked by *D.platura* in some regions of Mexico.
